# The Feeding of Infants and the General Practitioner*Read at the joint meeting of the Monroe and Livingston County Medical Societies, August 13th, 1914.

**Published:** 1914-11

**Authors:** C. G. Leo-Wolf

**Affiliations:** Buffalo, N. Y.


### BUFFALO MEDICAL JOURNAL

Yearly Volume 70. NOVEMBER, 1914 No. 4

### ORIGINAL ARTICLES

The right is reserved to decline papers not dealing with practical medical and surgical subjects, and such as might offend or fail to interest readers. Contributors are solely responsible for opinions, methods of expression and revision of proof.

### The Feeding of Infants and The General Practitioner.*

By C. G. LEO-WOLF, M. D.,

Buffalo, N. Y.

"Infant Feeding"—what a flood of memories this subject will tend to awaken in the mind of the general practitioner; of methods which have come and gone, of signal success and of dismal failures with anyone of these, of grateful parents and of little graves filled with blasted hopes.

You will surely ask me the question, who is responsible for this uncertainty, this insecurity which confronts the average medical man when he approaches the bedside of an infant suffering from some digestive disturbance?—and as I believe in fixing the blame where it really belongs, without mincing my words, I shall have to state to you that the ones responsible for this are the pediatricians, the writers of textbooks on the diseases of children.

If you will open almost anyone of the numerous books on Pediatrics under the head of infant-feeding, you will be struck by a remarkable similarity, in that page after page is filled with advice about the artificial feeding and comparatively few pages only deal with breast-feeding. May we not assume from this that these authors consider the unnatural bringing up of infants to be of greater importance than the natural method?

Such may be the case amongst the so called upper classes who closely approach in their mode of life the days of degenerate Rome, not of the Republic, but of the last two or three centuries of the Empire, when love of pleasure and ease

*Read at the joint meeting of the Monroe and Livingston County Medical Societies, August 13th, 1914.

drowned all sense of duty, when women had no time for their children and when they thought that they had done more than their duty when they had born a child and left its bringing up to menials.

Anyone who will take the trouble of investigating this subject will find that at least 90% of all mothers are able to nurse their offspring, and in some clinics even 95% succeed in doing so. Why then, do I ask of you, can it be possible that at the present time and in this country of ours not more than 40 or 45% of the women nurse their babies?

The explanation for this appalling condition is readily found. We physicians have not been doing our full duty, we have also been too credulous in accepting some flimsy excuse why the young mother could not nurse her child, though we knew full well that this was nothing but her thinly veiled unwillingness.

Many of these women are not vicious but only thoughtless. They do not realize of what they deprive their little ones in denying them what is theirs by nature, the only food which was ever intended to be placed into a baby's mouth, the milk of its own mother. They are misguided by what they read in the daily papers and in their magazines about the many ways in which they can change cows-milk to be equal to or even better than the product of their own breasts. They have little books, written by reputable pediatrists at that, which give all kinds of formulae, and last but not least in their baneful effect are the booklets put out by the manufacturers of proprietary baby-foods, which come by every mail after the birth of the child has been filed with the registrar of vital statistics.

Can this cndition be changed? Is it possible to educate the woman of today? Can we make her realize which way her duty points? We most surely can, if we will only take the time and trouble to explain matters to her and show her conditions in their true light. Women of today are better educated and more enlightened than they ever were before. Our State Department of Health has started a most wonderful and far-reaching educational campaign and it is ready to lend its aid to anyone who desires to make use of it.

When you are engaged to attend a pregnant woman in her imminent confinement or at your first call upon the puerpera talk these things over with her, reason with her and, if necessary, you may even paint to her in the darkest colors the possible consequences of unnatural feeding. Do not rest until in your practice the proper quota of women nurse their infants, this will be something for you to be proud of. Not only will you have the satisfaction of knowing that you have done your full duty towards those entrusted to your care but there is another, a more material side to this: 90% of nursing babies in your practice will mean 90% less trouble and worry and sleepless nights, not only for your patients but for yourselves as well.

In order to be able to succeed in this however you must thoroughly familiarize yourselves with the physiology of nursing, so thoroughly that you can successfully refute any argument brought up in favor of its discontinuation.

The most important point is this—to get the baby started right from the very first day of its life, this I have emphasized in my paper on "The Care of the New-born* which I read before the Pediatric Section of our State Society last year, where I advised that the baby be given nothing on the first day, that it be nursed twice during the second day, three times on the third, four times on the fourth and five times daily from the fifth day on, namely at 6 and 10 a. m. and at 2, 6, and 10 p. m. and nothing at night, this you will find most effective in every way and in most of your cases; the babies get plenty of nourishment and you take away all chance of their suffering from the most frequent source of trouble, namely overfeeding; the mother will also like it because it will not only give her time for her housework and for her social duties and diversion, but it will also insure for her sufficient sleep, lack of which is undoubtedly one of the most frequent causes for an insufficiency of the milk-supply.

One thing you must always remember, namely that in some cases, especially in primiparae and where the puerperae have been in poor health during gestation, the milk may not be present in sufficient quantities for days and even weeks, in some cases I have had to wait as long as six weeks and even longer before the mother had enough to satisfy her baby, just the same as I have succeeded in quite a number of cases in getting back a sufficient flow of milk where the baby had been taken off the breast for some invalid reason.

In these cases you will have to put the infant to the breast at the appointed hours and let it take what it can get and then, but then only, give it some additional food with the bottle. What to give in the bottle I shall tell you later when I come to the unnatural feeding of infants. How much you have to give can only be determined by careful weighing before and after each nursing, and then giving only so much as to make up the actual deficiency, because the danger of overfeeding is great in this kind of allaitement mixed.

Some infants are either too lazy or too weak to exert enough power to act as a stimulus to the breasts, in these cases it is a good plan to take a wet-nurse into the house for a few weeks with her baby; you let the weak baby nurse at the breasts of the wet-nurse, while her strong baby sucks at the breasts of

---

*Care of the New-born, Archives of Pediatrics, May, 1914.

your patient thus supplying sufficient stimulation to cause their increasing secretion whilst it makes up the deficiency of its nourishment at the breasts of its own mother. As soon as the breasts of your patient furnish a sufficient supply and her baby is strong enough to draw it you may dismiss the wet-nurse.

This method of wet-nursing is very efficient in a great many cases in which we would otherwise have had to have resort to outright wet-nursing, which is inhumane to a certain extent, as it deprives the infant of the wet-nurse of its own rightful food, and in many cases leads to the same dangers from which we try to save our little patient, or in which we would have had to resort to the bottle and its consequences.

If an apparently healthy infant does not thrive at the breast you should always think first of all of congenital syphilis, which frequently does not put in appearance until about the fourth month of life, or the signs of which may be so slight as to be easily overlooked by one who is not very familiar with these. The taking of a careful family history will be of great aid in these cases, as will also be a Wassermann taken from the blood of the mother or the child, or both, though I have seen quite a number of cases with the clear symptoms of congenital lues in which the Wasserman reaction did not become positive until after some months had elapsed. In cases in which you can safely rule out this disease do not waste your time with the old fashioned pseudoscientific methods of examination of the breast-milk. It may look very learned and make some impression on your patient to ask her to express some of her milk into a test-tube so that you can take it home to examine it under the microscope, but you will only be fooling yourselves and your patients.

Think it over and you will realize at once that you are only examining that particular sample, which may contain much or little cream according to the time it was drawn; if it was drawn before the baby had nursed it will contain little cream, if after the nursing much, it will also make a big difference if it was drawn after a nights rest or in the evening; according to these circumstances the contents of cream in a woman's milk may vary from one to six per cent. of cream at different times during one and the same day.

If you really desire to make an examination of the milk as to its contents, then you must ask for samples of equal quantities, say 5 c. c. drawn before and after each and every nursing during one day, mix these and then examine the same as you would test cows-milk, and in order to get figures which would be of some value you should do this for three consecutive days, so as to eliminate the error accruing from possible daily variations which in some cases may be quite considerable.

If you desire to know how much milk the baby is getting from the breasts, you can easily find this out by having the baby weighed before and after each and every nursing for at least three consecutive days and adding the daily amounts; but do not use spring scales for this as they are entirely too inaccurate for this purpose.

One thing which has always seemed most incongruous to me is this, that every time a baby does not thrive at the breast the physician is very liable to think that something is wrong with the milk itself and consequently advises weaning. Could not something be wrong with the method of nursing? How many times have you instructed the young mothers how to do this and which is the easiest and best way and which the most comfortable position? Some women have severe backache every time they attempt to nurse which will disappear when they assume the right position; some have sore nipples which cause them considerable pain and a reflex holding back of the milk, a few applications of anaethesin until the nipples are healed will easily remedy this. Others again have small nipples and will be successful after we have taught them to place part of areola in the baby's mouth as well. Or on the other hand the fault may lay with the infant which also has to be taught; some do not learn until late how to nurse; others may have some nasal obstruction or a slight coryza and a drop of adrenalin may remedy this condition and thus lead to success.

One point we must never forget, namely that primiparae especially may be worrying about their ability to nurse and get nervous about it, all we have to do in these cases is to reassure these women and to give these the necessary advice, and if we do not succeed with this alone we may resort to one of the so called lactagoga, which however act through their mental effect only and are no better to increase the amount of milk than good everyday home-cooking, with plenty of variety and the things the patient always liked, but not some fanciful diet which is based mainly on some old and long controverted superstitions. Every kind of good and nourishing food taken in moderation but still in sufficient amounts which is eaten with relish will without any unfounded restrictions have the desired result.

So much about breast-feeding, we will now consider briefly what we can do for the 10%, the poor unfortunates who are denied their birthright.

Whenever the general practitioner is face to face with the problem of the unnatural feeding of an infant he approaches the question what to give with fear and trepidation, and this is by no means to be wondered at; let him open any two of the textbooks on diseases of children or on infant-feeding and he will notice at once the great diversity of opinion on this subject.

How can this be explained? Can it be possible that Prof. A. has splendid results with certain percentages of cream, sugar and proteids, whilst Prof. B. has seen nothing but failures from this first method and therefore has worked out a method of his own on diametrically opposite principles? One explanation for this, and to my mind the most plausible one, is this, that some infants will thrive on almost anything in the line of food whilst others flatly refuse to adapt themselves to even the most wonderful and seemingly the most scientific formulae.

You have all heard and read a great deal of late about percentage feeding, about caloric feeding, about top-milk mixtures, about cream mixtures and what not else and you will naturally ask me which one of these systems do you believe in and which one do you advise us to follow? I shall tell you do not use any of these, but use what the Lord has given to you—COMMON SENSE. Do not feed formulae but good sensible combinations of food elements, do not feed by rule of thumb or by published formulae, or by the marks on a glass jar, or some ingenious rotary indicator; remember always that you have to deal with individuals, infants though they be, who have their likes and dislikes and their idiosyncrasies the same as you have, which may be either inherited or acquired, and that you have not got to deal with pieces of machinery where you can say beforehand that you will need so much fuel or so many volts to get the expected result. If you will always keep this before your mind and refrain from any rigid system in feeding infants you will succeed in most of these cases, provided you keep in mind also the few general rules I am about to give you:

The key-note of successful infant-feeding is to remember that the infant requires three ounces of liquid, mind liquid not milk—for every pound of its weight up to one quart, that is when its weight will have gone up to eleven pounds and that you must never give more than one quart.

Remember further than in order to thrive and grow the infant requires forty calories per pound of its weight, but as I have warned you before, find out first what the baby's digestive apparatus will stand, and then figure out for your own satisfaction if this food represents the necessary quantity of calories.

In summer the infant requires more water, the same as we all do, but not too much because this excess would have to be excreted, not as plain water but as urine which is a solution of salts of which the body would consequently be deprived.

Get away from the old and worn idea that summer is the worst time for the baby, and that it is at this season only that we see the digestive troubles in these; the moist heat of the summer months is only the last straw in an already impaired system, the foundation for which was laid during the other months. I see more of these cases in consultation in winter when the confinement in the bad air of our overheated and underventilated homes exerts its bad effect.

Of the elements of food protein is the least dangerous, even in large quantities.

The carbo-hydrates can be given up to 10%, maltose is safer to add to the food than either lactose or canesugar, and we have learned from experience that two carbo-hydrates act better than one. Overfeeding with carbo-hydrates leads to a dangerous condition of retention of water in the body which is very unstable and which may be lost inside of a few hours and thus cause the sudden and severe losses in weight which we see in the condition still called cholera infantum.

Fat I consider the most dangerous element of the food, and we must always be extremely careful with its administration and for this reason I must warn you against topmilk and still more against cream mixtures. I have for some years advised starting from fat-free bases, such as skimmilk or buttermilk, whenever I have taken charge of infants with digestive troubles and had the best of results, on the other hand I have seen many an infant in whom a pronounced idiosyncrasy against cream had been brought on by the injudicious administration of this

Remembering also that it always is safer to underfeed an infant, at least until we have had time to find out what its digestive apparatus can take care of, rather than to start in with full quantities and then to learn to our great chagrin that we have lost valuable time and that we then have to begin again where we should have started in the first place.

As to the patented and proprietary foods which are so numerous and the blatant advertising of which makes our work so difficult, I want to ask you to beware; do not use any of these; not one of these is any better than what you can do with what you can find or easily procure in any well regulated household and much cheaper at that, and you must not forget that the expense is a great factor in most of the families with which we have to deal. Who do you think pays for the advertising in the lay and the medical press, and for the "wonderful" illustrated booklets sent out broadcast if not the consumer?. Do you think for one moment that the manufacturer is in this business for humanitarian reasons and not for the money that he can extract from the pockets of the unwary? If you should be taking care of a case of typhoid or pneumonia, do you leave some patent medicine with a booklet of the respective disorder and tell the family to read it and then treat the patient according to the information contained in the folder accompanying the package or on the label? Of course not; why then should you let the ignorant and greedy manufacturer teach you how to feed the babies?

One more point touching upon the present crusade for pure milk and the movement on foot to have all the milk sold for infant-feeding pasteurized. It is one thing to say that we must give our infants as good and as clean a milk as possible, one that is free from pathogenic germs, but it is quite another thing to persist in blaming the bacterial count of the milk for the damage which is wrought by the fanciful foolish mixtures we have been in the habit of feeding. A very small minority of the cases of so-called summer-complaint in infants is caused by these bacteria and I have seen many a case of marasmus in children who had never had anything but the most expensive and cleanest milk from special dairies. Intestinal tuberculosis from bovine disease is now recognized to be extremely rare.

True, I advise boiling the milk, but this I do not on account of any desire on my part to kill the bacteria, but because it has been found that boiling makes the proteins of cows milk easier of digestion.

With these simple rules you will succeed in the majority of the 10% which you have to bring up unnaturally, the few that will be left offer such serious problems that you will best leave the worry about these to the pediatrist who, owing to his special training, will find means and ways which might not occur to you as general practitioners.

From about the sixth month of life I advise that the baby be given one feeding daily of some pap or some soup in which all the vegetables in season may be cooked and strained, this latter especially in anemic infants, this to take the place of one nursing or one bottle, and I also advise that a little orange juice, one or two teaspoonfuls, be given one hour before the second and the fourth feeding. Later during the seventh and eighth month I supplant one other feeding with pap or soup, and this will prepare the child for the time of weaning which is to be instituted about the end of the ninth month.

What to feed the baby after this, I have told in another paper* which is to be published soon.

481 Franklin Street.

---

*The Dietetic Treatment of Children during the second period of life, to be published soon in the Medical Record.

